# SOCS家族分子在肿瘤发生发展中的作用

**DOI:** 10.3779/j.issn.1009-3419.2016.09.11

**Published:** 2016-09-20

**Affiliations:** 1 300052 天津，天津医科大学总医院肺部肿瘤外科 Department of Lung Cancer Surgery, Tianjin Medical University General Hospital, Tianjin 300052, China; 2 300052 天津，天津市肺癌研究所，天津市肺癌转移与肿瘤微环境实验室，天津医科大学总医院 Tianjin Key Laboratory of Lung Cancer Metastasis and Tumor Microenvironment, Tianjin Lung Cancer Institute, Tianjin Medical University General

**Keywords:** 细胞信号转导抑制分子, JAK-STAT, 甲基化, Suppressor of cytokine signaling (SOCS), JAK-STAT, Methylation

## Abstract

细胞因子信号转导抑制分子（suppressor of cytokine signaling, SOCS）是一类在细胞信号转导过程中发挥重要作用的负调控因子，家族成员一共有8个，主要通过抑制JAK-STAT信号通路的持续激活而调节细胞的增殖、分化和凋亡。在肿瘤的发生发展过程中，由于SOCS家族基因启动子区CG岛超甲基化、组蛋白异常乙酰化、基因突变、基因缺失等原因导致的SOCS蛋白表达异常，使JAK-STAT被持续活化，从而导致肿瘤的发生发展与转移。本文综述了SOCS家族的发现，成员构成及分子结构，各结构域的功能及其在肿瘤发生发展等方面的最新进展。由于SOCS在肿瘤发生、发展方面的重要作用，SOCS分子作为信号转导途径重要的负调节因子发挥肿瘤抑制作用，使其成为肿瘤治疗的新靶标。

## Abstract (en)

Suppressor of cytokine signaling (SOCS) family proteins are a group of negative regulatory factors that plays important roles in the negative regulation of cytokine responses by terminating the activation of the JAK-STAT and other signaling pathways. The family is composed of eight structurally related proteins. mainly through the inhibition of the activation of JAK-STAT signaling pathway and regulates cell proliferation, differentiation and apoptosis. In the process of tumor progression, the promoter CG island hypermethylation, gene mutation, gene deletion and inactivation lead to the abnormal expression of SOCS protein make JAK-STAT continuous activation, resulting in the development and metastasis of tumor. Here, we review the SOCS family members found, composition and molecular structure, the domain of the function, and the latest progress of development in tumor. Based on the important role of SOCS in tumor development, SOCS as a negative regulator factor represent a kind of tumor suppressor genes, has become a new target for tumor therapy.

细胞因子（cytokine, CK）是免疫原、丝裂原或其他刺激因子诱导多种细胞产生的低分子量可溶性蛋白质，具有调节天然免疫和获得性免疫、血细胞生成、细胞生长以及损伤组织修复等多种功能。他们通过与细胞表面相应的受体特异性结合，从而将细胞外的信号通过不同的途径传递至细胞内^[1, 2]^。细胞因子与其细胞表面相应的受体结合，激活受体相关的JAK蛋白酪氨酸激酶（Janus protein tyrosine kinase, JAK）的激酶，使位于下游的信号传导及转录激活因子（signal transducers and activators of transcription, STATs）C端的保守酪氨酸残基发生磷酸化而使之得到激活。被激活的STATs通过其酪氨酸磷酸化的SH2结构域（Src homology domain）相互结合形成二聚体，进入细胞核，与特定的DNA位点结合，从而调控靶基因的转录。现有的证据表明，JAK-STAT信号通路的持续性活化与肿瘤的恶性转化关系密切，并且与肿瘤的发生、发展有关。而导致其持续活化的机理，一直是研究的热点。1997年Starr、Naka和Endo等^[3]^学者首次发现了细胞因子信号转导抑制（suppressor of cytokine signaling, SOCS）蛋白，并首次阐明*SOCS*基因对JAK-STAT信号通路的负调控作用，之后的研究^[4, 5]^发现，SOCS家族在肿瘤发生、发展和转移等过程中发挥着重要作用，而日益成为研究关注的焦点。近年来研究发现，SOCS启动子区域内CpG岛（CpG island）的超甲基化、组蛋白乙酰化异常、突变及其失活导致的基因转录沉默与多种肿瘤的发生密切相关。SOCS蛋白作为信号转导途径的负调节因子成为治疗肿瘤的新靶标。本文就SOCS家族的发现、结构特点、各结构域的功能及其在肿瘤发生发展中的作用等方面的研究进展作一综述。

## 1 SOCS家族的分子结构

SOCS1于1997年从IL-6诱导小鼠单核白血病细胞MI系的分泌体系中发现，以后又陆续发现了其他SOCS蛋白，目前发现该家族有CIS（cytokine-inducible SH2 domain-containing protein）、SOCS1-SOCS7等8个成员分子^[3, 6, 7]^，SOCS家族成员及其分子结构概括列于图 1。SOCS家族可在多种细胞内构成表达或诱导表达，不同种系的同一种SOCS的同源性高，表现为高度保守。

**1:**
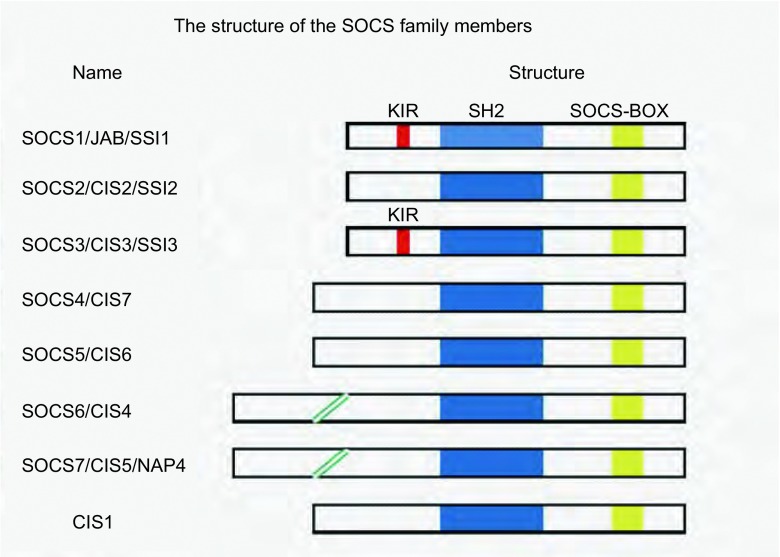
SOCS家族成员的分子结构图。SOCS家族各成员均包含一个SOCS盒和一个中心SH2同源区。而在SOCS1和SOCS3的中心SH2区上游区段还包含一个特有的KIR区。 The structure of the SOCS family members. All the members contain a SOCS box and a central SH2 homology region. Both SOCS1 and SOCS3 contain a unique kinase inhibitory region (KIR) on the upstream of the central SH2 domain, which is proposed to function as a pseudosubstrate.

SOCS家族的蛋白结构类似，均由N区、SH2区和C端的SOCS盒（SOCS box）区组成。中间的SH2区含SH2结构域，其协同N区，通过与特定细胞因子受体结合使不同的SOCS蛋白识别不同的目标，进而调节各种细胞因子信号转导途径。SOCS盒由近40个氨基酸组成，同源性极强，具有高度保守性，SOCS盒通过与Elongins B、Cullin-5或Cullin-2、Rbx1和E2相互作用使该区域的蛋白分子作为E3泛素连接酶（ubiquitin ligase E3）降解与其结合的特异性信号蛋白，同时抑制自身蛋白的降解，从而调节细胞内信号分子的定位或稳定性，抑制细胞信号转导^[8-10]^。此外，SOCS1和SOCS3可以通过其激酶抑制区直接抑制JAK激酶的活性，激酶抑制区的功能是作为假底物，被认为是SOCS1和SOCS3执行其负反馈调节必不可少的区域^[11]^。最近，Babon等^[12]^的研究表明其并不仅表现为假底物，还能作为一个非竞争性抑制剂与ATP（phosphate donor）和底物结合。SOCS家族各成员发挥作用的方式有所不同，SOCS1的SH2结构域能够直接结合JAKs活化环^[11]^，CIS和SOCS2的SH2结构域的酪氨酸残基能够发生磷酸化，SOCS3可以直接结合活性细胞因子受体^[13]^，与SOCS分子结合的酪氨酸磷酸化蛋白质包括TLR（toll-like receptor, TLR）信号^[14]^和人胰岛素受体底物1（insulin receptor substrate 1 antibody, IRS1）^[15]^。因此，SOCS蛋白通常通过SH2结构域结合，通过SOCS盒泛素化诱导靶分子的降解。而且有研究^[16]^发现其他含有SOCS盒区的非SOCS家族成员的蛋白也可以通过SOCS盒的作用来调节肿瘤细胞的侵袭和转移。

## 2 在肿瘤发生发展中的作用

细胞因子信号通过抑制JAK-STAT信号通路介导的信号转导在各种类型的细胞增殖、分化、成熟和凋亡过程起着重要的作用，并参与肿瘤的发生和发展。大量的细胞因子可以激活JAK-STAT信号通路，如白细胞介素6（interleukin-6, IL-6）、白细胞介素11（interleukin-11, IL-11）、α干扰素（interferon-α, IFN-α）、粒细胞集落刺激因子（granulocyte colony-stimulating factor, G-CSF）、白血病抑制因子（leukemia inhibitory factor, LIF）和瘦素等，这些细胞因子与相应的受体结合后导致受体发生二聚化，JAK激酶在接受上游受体分子的信号后，迅速募集于受体上并发生活化，活化的JAK催化受体发生酪氨酸磷酸化，通过磷酸化STAT分子SH2的识别和结合位点，STAT形成二聚体并进入胞核。二聚体STAT分子作为有活性的转录因子，直接影响相关基因的表达，进而改变靶细胞的增殖或分化状态^[17]^。在肿瘤细胞中JAK-STAT被持续活化，从而促进肿瘤细胞的增殖和分化。SOCS分子是经典的JAK-STAT信号通路的负反馈调节蛋白^[18]^，其可以通过抑制STAT的磷酸化及其二聚体的形成或者直接抑制JAK的磷酸化来负向调节JAK-STAT通路，从而抑制细胞的持续增殖和分化。SOCS失活后其对JAK-STAT通路的抑制作用也随之消失，从而导致肿瘤细胞的持续增殖和分化（信号转导的过程见图 2）。

**2:**
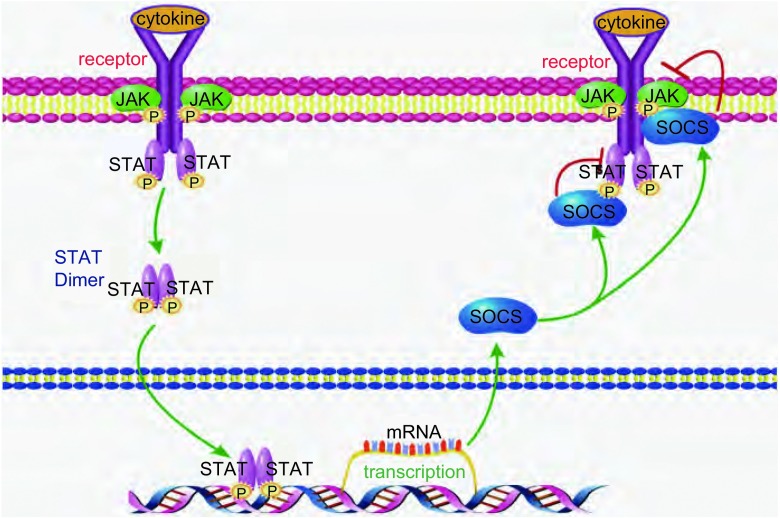
SOCS抑制JAK-STAT信号通路的作用机制 Mechanism of SOCS inhibition of JAK-STAT signaling pathway

## 3 肿瘤中SOCS失活的机制

在肿瘤细胞中SOCS可由于多种原因失活，导致其失活的可能机制有以下几种。

### 3.1 超甲基化

癌细胞中*SOCS*基因沉默与其启动子CpG岛的超甲基化密切相关。由于SOCS启动子区的异常甲基化，SOCS蛋白在许多肿瘤细胞中的表达降低，导致其对JAK-STAT信号通路的抑制作用消失，STAT持续被磷酸化激活，从而导致肿瘤的发生。

SOCS超甲基化在多种肿瘤中被发现，如：肺癌、肝癌、前列腺癌、食管癌等^[19-22]^。Souma等^[23]^学者在胃癌的研究当中发现，在其所选择的5种胃癌细胞中均有SOCS1的甲基化存在，并且其中的两个细胞系当中有STAT3的组成性激活，当使用腺病毒载体来增加SOCS1的表达后，其STAT3磷酸化水平明显降低，同时也抑制了细胞的异常增殖。他们还发现SOCS1对肿瘤细胞的抑制作用不仅仅是因为抑制STAT3的活化，还通过对P38-MAPK（mitogen activated protein kinases）的抑制作用来抑制细胞恶性增殖。在异种移植瘤的模型当中增加SOCS1的表达能够明显抑制胃癌的增殖，从而证实了*SOCS1*基因甲基化在胃癌发展过程中的关键作用。Takeuchi等^[24]^亦证实SOCS超甲基化在儿童淋巴癌中起了关键性的作用。Kim等^[25]^发现在宫颈癌中SOCS1的表达降低同样与*SOCS*基因启动子区的甲基化相关。

在肝癌的研究当中，对不同临床病理特征的病例与其SOCS1启动子区甲基化之间的关系进行对比分析，发现SOCS1启动子区甲基化与肝硬化源性的肝癌和肿瘤的大小之间存在明显的相关性^[26]^。日本的学者用实时定量PCR和免疫印迹的方法发现肝癌组织中SOCS3的表达低于正常肝组织，并且通过特异性敲除小鼠SOCS3基因，使得SOCS3对STAT3的抑制作用消失导致STAT3持续活化，其抗细胞凋亡的作用随之增强，致癌物诱导肝癌发生的作用也随之增加，证明了超甲基化导致的SOCS3表达缺失与肝癌的发生有关^[27]^。英国学者最新研究发现，SOCS3缺失能够明显促进骨髓源性肿瘤的生长，这主要是通过增加肿瘤微环境中相关髓源性抑制细胞和减少CD8+T细胞的浸润而实现的，重要的是，他们还发现粒细胞集落刺激因子在该途径当中起着重要作用，表现出STAT3的异常活化并优先分化为Gr-1^+^CD11b^+^Ly6G^+^MDSC表型。而骨髓源性肿瘤中SOCS的表达缺失也与SOCS启动子区的甲基化有关^[28]^。Lindemann等^[29]^发现，在原发性胶质母细胞瘤中SOCS3启动子甲基化和表皮生长因子受体的表达呈负相关，他们发现在沉默SOCS3之后，表皮生长因子受体相关信号通路被激活，进而增加了STAT3和局部粘着斑激酶（focal adhesion kinase, FAK）的磷酸化水平，证实了SOCS3启动子甲基化失活通过STAT3和FAK活化促进胶质瘤细胞的侵袭转移作用。Coppede等^[30]^研究发现结肠癌中SOCS的CpG岛甲基化可以作为一种重要的分子标记。

### 3.2 组蛋白乙酰化

SOCS的表达除了与甲基化有关以外，与组蛋白的去乙酰化也有一定的关系。Kim等^[25]^在宫颈癌中发现SOCS1的表达降低不仅与其启动子区高甲基化有关，同时还发现SOCS1和SOCS3的表达与组蛋白的去乙酰化有关，用选择性组蛋白去乙酰化酶处理之后，二者的表达均增高，而SOCS5的表达没有明显的变化。在结直肠癌的研究当中也表明组蛋白乙酰化参与了SOCS1和SOCS3的表达调控，组蛋白去乙酰化酶（histone deacetylase, HDAC）抑制剂能够导致SOCS1和SOCS3启动子相关的组蛋白乙酰化，使得SOCS1和SOCS3的表达增高而抑制JAK-STAT通路的信号转导，继而调节肿瘤细胞的增殖分化和凋亡^[31]^。因此，SOCS启动子区组蛋白乙酰化可能也与肿瘤的发生发展有一定的关系。

### 3.3 突变Mottok等^[32]^发现SOCS的表达下调与异常的特异

性体细胞突变（somatic hypermutation, SHM）有关，在人类弥漫性大B细胞淋巴瘤、滤泡性淋巴瘤中*SOCS1*基因的突变率约为四分之一，这些突变是基于B细胞特异性体细胞突变引起的。髓系细胞*SOCS3*基因缺失通过使肿瘤微环境中髓源性抑制细胞水平明显升高并降低CD8^+^T细胞的侵润，使得JAK-STST通路持续激活从而促进肿瘤的生长^[28]^。有作者在胃癌的研究当中发现，SOCS6在胃癌当中表现为持续的下调，他们的研究数据也表明SOCS6的下调主要与等位基因的突变缺失和启动子甲基化有关，后来他们还证实了SOCS6诱导的细胞凋亡与线粒体的分裂有关^[33, 34]^。SOCS负性调控JAK-STAT通路进而抑制肿瘤的增殖和生长，STAT信号通路的激活促成了肿瘤炎性微环境的形成，参与了肿瘤血管生成、上皮间质转化和细胞外基质降解等多个环节，在肿瘤的侵袭和转移过程中发挥重要作用。突变导致的SOCS失活后，其对JAK-STAT信号转导通路的抑制作用也随之消失，这就使得JAK-STAT信号通路的持续激活，促进肿瘤微环境的形成，最终导致肿瘤的发生发展和转移。

## 4 SOCS与病毒感染相关的肿瘤

据估计至少有20%以上的肿瘤是由炎症发展而来，如肝癌患者当中多数都是由HBV或HCV病毒感染导致的肝炎、肝硬化发展而来^[35]^。在这些肿瘤组织中SOCS1基因CpG岛处于超甲基化状态，导致SOCS1蛋白的表达降低，Yoshida等^[26, 36-38]^观察到，许多肝炎病毒感染的病例在发展成为肝癌之前已经出现了*SOCS1*基因的沉默，并且SOCS1启动子异常甲基化与肝炎病毒的持续感染有关。SOCS1的表达降低会导致STAT1的过高表达，加重肝组织的炎症反应，促进肝细胞转化为上皮细胞并持续增殖，而增加恶化的可能性。因此，SOCS是一个通过抑制慢性炎症而防止恶变的独特的抑癌基因，将来可以作为一个防止组织炎症向肿瘤转化的治疗位点^[39, 40]^。

## 5 研究SOCS家族功能的意义

自SOCS家族第一个成员被发现以来，人们对其进行了大量的研究，以了解SOCS蛋白家族成员的分子结构及其功能，以及其对JAK-STAT通路的负调控作用在肿瘤的发生发展中起的作用。众所周知，癌症是目前人类面临的最大的医学难题之一，手术、放射治疗、化疗是传统的治疗方式。放射及化学治疗途径对肿瘤细胞的杀伤不完全，并存在着选择性低、毒性大、免疫抑制等缺点。近些年来，随着人们对肿瘤发生发展机制认识的加深和基因检测手段的不断提升，使得靶向药物治疗成为了肿瘤方面的研究热点。现在有很多种靶向药物已经用于临床，使很多肿瘤患者从中获益。靶向药物具有针对性强、对正常细胞毒性小、效果明显的特点，这就为很多因难以耐受传统化疗药物毒副作用的患者提供了新的选择。SOCS蛋白在肿瘤发生发展中的作用正在逐渐的被人们认知清楚，现在已经有许多学者将SOCS蛋白激活剂用于临床前研究，并取得了一定的进展，有研究指出肿瘤的治疗中激活SOCS后再用JAK抑制剂的效果比单用JAK激酶抑制剂的效果要好^[23, 41-43]^。Kim等^[25, 42, 44]^学者发现恢复SOCS1和SOCS3的表达能够增加宫颈癌细胞对放疗的敏感性。在非小细胞肺癌的研究当中也发现增加SOCS1的表达能够增加P53的磷酸化水平，而在仅使用JAK抑制剂时却没有P53的磷酸化，恢复SOCS3的表达不仅能够促进肿瘤细胞的凋亡，而且还能增加其对放疗的敏感性。Al-Jamal等^[45]^发现在慢性粒细胞性白血病的治疗中，*SOCS3*基因启动子区的甲基化所导致的STAT3持续激活与肿瘤细胞对伊马替尼的耐药有关。

综上所述，SOCS家族成员是JAK-STAT信号通路重要的负调控因子，由于超甲基化、组蛋白乙酰化、突变等原因导致其失活，是肿瘤发生、发展的重要分子机制。SOCS家族的激活能增加肿瘤细胞对放疗、化疗药物及靶向治疗的敏感性，并诱导肿瘤细胞凋亡。针对SOCS的靶向治疗的研究，将会为肿瘤靶向治疗提供新的思路。
